# Diabetes Mellitus and Cardiovascular Diseases: Nutraceutical Interventions Related to Caloric Restriction

**DOI:** 10.3390/ijms22157772

**Published:** 2021-07-21

**Authors:** Pamela Senesi, Anna Ferrulli, Livio Luzi, Ileana Terruzzi

**Affiliations:** 1 Department of Biomedical Sciences for Health, Università degli Studi di Milano, 20131 Milan, Italy; pamela.senesi@unimi.it (P.S.); anna.ferrulli@unimi.it (A.F.); livio.luzi@unimi.it (L.L.); 2 Department of Endocrinology, Nutrition and Metabolic Diseases, IRCCS MultiMedica, Sesto San Giovanni, 20099 Milan, Italy

**Keywords:** diabetes, cardiovascular diseases, caloric restriction, berberine, resveratrol, quercetin, L-carnitine

## Abstract

Type 2 diabetes (T2DM) and cardiovascular disease (CVD) are closely associated and represent a key public health problem worldwide. An excess of adipose tissue, NAFLD, and gut dysbiosis establish a vicious circle that leads to chronic inflammation and oxidative stress. Caloric restriction (CR) is the most promising nutritional approach capable of improving cardiometabolic health. However, adherence to CR represents a barrier to patients and is the primary cause of therapeutic failure. To overcome this problem, many different nutraceutical strategies have been designed. Based on several data that have shown that CR action is mediated by AMPK/SIRT1 activation, several nutraceutical compounds capable of activating AMPK/SIRT1 signaling have been identified. In this review, we summarize recent data on the possible role of berberine, resveratrol, quercetin, and L-carnitine as CR-related nutrients. Additionally, we discuss the limitations related to the use of these nutrients in the management of T2DM and CVD.

## 1. Introduction

### 1.1. Diabetes and Cardiovascular Diseases

The prevalence of type 2 diabetes mellitus (T2DM), the most common form of diabetes, has rapidly increased and currently represents a devastating socioeconomic burden worldwide.

The diabetic condition is a crucial risk factor for the onset of cardiovascular diseases (CVD), including retinopathy, stroke, and cardiac damages [1]. T2DM is linked to coronary heart disease, myocardial stroke, and diabetic cardiomyopathy [2,3].

It is important to point out that T2DM and CVD mutually strengthen each other, increasing the hospitalization rate and mortality. High glucose levels and in particular glucose fluctuations impair energy production, excitation, and contraction of cardiac cells, favoring the onset of several cardiac pathologies, including atrial fibrillation. In addition, hyperglycemia modifies cardiac intracellular pathways, increasing the synthesis of reactive oxygen species (ROS) and inflammatory mediators [4,5,6]. Oxidative stress impairs the capacity of heart cells to respond to injuries: the effectiveness of traditional and innovative cardiac treatments aimed at recovering myocardial function after infarction is limited in diabetes patients [3,7,8]. Moreover, chronic hyperglycemia, increasing ROS production, leads to the development of endothelial dysfunction that significantly contributes to the pathogenesis of micro- and macrovascular diseases [9].

**Figure 1:**
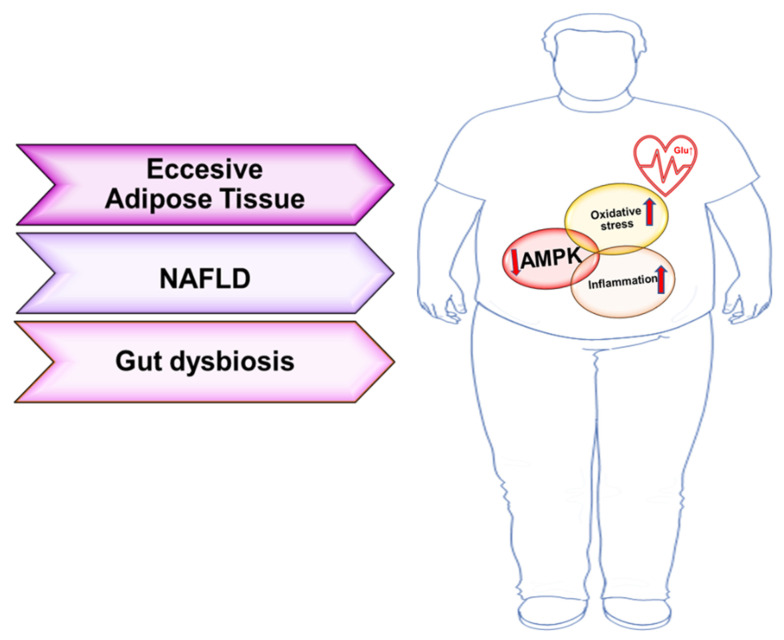
Schematic representation of the strong interconnection between T2DM and CVD. Excessive adipose depots, gut dysbiosis, and NAFLD are key risk factors that promote T2DM and CVD. At the molecular level, oxidative stress and inflammatory condition are the primary mediators of metabolic and cardiac damages. These processes are orchestrated by AMPK impaired signaling (modified by Servier Medical Art by Servier is licensed under a Creative Commons Attribution 3.0 Unported License).

Not only oxidative stress but also chronic low-grade inflammation is a key pathological mechanism involved in T2DM and CVD [9,10]. Excessive depots of adipose tissue and microbiota dysbiosis cause a pro-inflammatory microenvironment [10,11,12] (Figure 1). White adipose tissue is characterized by improved macrophage infiltration that is associated with increased systemic insulin resistance condition. Moreover, adipose tissue, as an endocrine organ, secretes different adipokines and above all several inflammatory mediators, including tumor necrosis factor (TNF)-α and interleukin (IL)-6, that play a crucial role in systemic low-grade inflammation state [10]. It is important to note that recent investigations have demonstrated that adipokines interacts with myokines modulating cardiovascular function. For example, apelin, an adipokine, ameliorates hypertension and endothelial dysfunction and counteracts infarct damages, decreasing ROS production [13,14].

Impaired composition and reduced diversity of gut microbial community are correlated with a decreased insulin sensitivity and worsening of the inflammatory state [15,16,17]. An unbalanced ratio of Firmicutes/Bacteroidetes, the two principal phyla of gut microbiota, damages the gut mucosal barrier and thus increasing the translocation of lipopolysaccharide (LPS), which is a component of Gram-negative bacteria, as well as contributing to the activation of inflammatory pathways, including Toll-like receptor 4 [18,19]. The subsequent abnormal production of cytokines exacerbates the inflammatory state in T2DM patients.

Moreover, gut dysbiosis plays a crucial role in non-alcoholic fatty liver disease (NAFLD), which is another typical comorbidity in T2DM patients with or without CVD [20] (Figure 1). Gut and liver are closely connected not only through the portal vein; accumulating data indicate that microbiota metabolites are capable of stimulating an important inflammatory response in liver, principally by Kupffer cells activation. Indeed, NAFLD patients are characterized by increased LPS levels [21]. In addition, gut metabolites increase insulin resistance and fat accumulation. Clinical studies have shown that the production of short-chain fatty acids by the microbial fermentation of dietary fibers is impaired in NAFLD subjects, inducing imbalance between energy harvest, expenditure, and mitochondrial dysfunction. The consequent overproduction of ROS and cytokines increases the risk of CDV onset in diabetic condition [22,23,24].

Lifestyle modifications, diet and physical activity, represent a primary cornerstone in the prevention and management of T2DM and CVD [25]. In this review, we will discuss emerging dietary interventions and principal food bioactive molecules that could represent an adjuvant therapeutic approach in T2DM/CVD management.

### 1.2. New Dietary Interventions in T2DM/CVD Management

The traditional nutrition approach is aimed at defining the correct intake of macronutrients and micronutrients to maintain ideal body composition and function [26], and consequently, traditional nutrition research, based mainly on epidemiological studies, has drawn up the dietary guidelines for the population.

In the last decades, sequencing of the human genome, “omics” technologies (genomics, transcriptomics, proteomics, and metabolomics) and progress in microbiota knowledge have allowed to make a Copernican revolution in nutrition research from “dietary for the population” to “personalized nutrition” [27,28]. Using “omics” technologies, several molecular events, i.e., pathways activation, epigenome stability, and protein expression, caused by nutrients intake have been identified. Now, the innovative goal of nutrition research is focused on identifying the relationship between food constituents and molecular targets [29,30]. This new approach of nutritional intervention, based on the molecular action of food and not only on nutritional value of food, represents the crucial therapeutic strategy in the T2DM and CVD. 

### 1.3. From Caloric Restriction to Caloric Restriction Mimetics

For T2DM patients, reduced daily calorie intake is the gold standard of dietary therapeutic interventions [31]. Studies performed using different animal models and human clinical trials have demonstrated that caloric restriction (CR), a nutritional approach based on caloric intake decrease (between 20 and 40%) without modifying the balance of micro/macronutrients, ameliorates cardiometabolic conditions and extends lifespan [30,31]. 

The CALERIE™ (Comprehensive Assessment of Long-term Effects of Reducing Intake of Energy) study is the first clinical trial focused on CR action [32]. It was organized in two different phases: in the first phase, different grades of CR (20%, 25%, and 30%) were tested in overweight subjects for six months to one year. In the second part of CALERIE™, constituted by a multicenter, randomized controlled trial, CR (25%) action was studied in healthy non-obese subjects for two years. Extensive parameters analyzed on different biological sample (serum, plasma, urine, and biopsies from skeletal muscle and adipose tissue subcutaneous abdominal) have demonstrated that after 2 years of CR, all cardiometabolic risk factors have decreased compared to baseline. In addition, metabolic parameters, including insulin sensitivity index and metabolic syndrome score, have ameliorated relative to control [32,33,34].

At the molecular level, AMPK (AMP-activated protein kinase) is the crucial mediator of CR effects enhancing insulin-sensitizing action and consequently glucose uptake in skeletal muscles and decreasing hepatic glucose production and improving fatty acid oxidation [35,36,37]. Notably, AMPK improves healthspan and lifespan, as demonstrated by in vivo studies performed using CR diet or metformin, which is the most common drug to treat TDM2 and is capable of active AMPK signaling [38,39,40,41]. 

**Figure 2:**
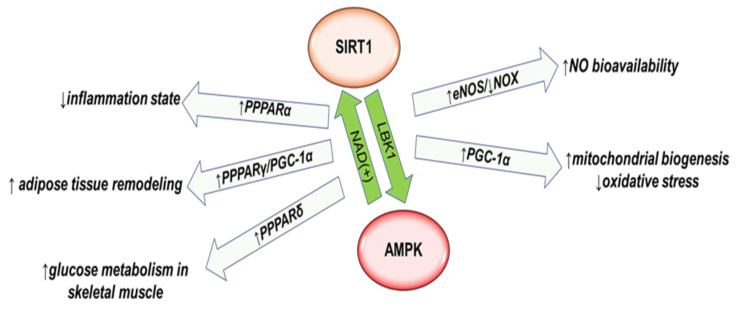
T2DM and CVD: AMPK–SIRT1 signaling cascade. AMPK, the main mediator of CR action, synergically acts with SIRT1. AMPK actives SIRT1, increasing NAD^(+)^levels, while SIRT1 promotes AMPK activity by Liver Kinase B1 (LKB1). AMPK/SIRT1 regulating the eNOS/NOX ratio increases NO bioavailability and mitigates endothelial dysfunction. Moreover, AMPK and SIRT1 activated PGC-1α, which is the primary factor involved in mitochondrial biogenesis. Then, AMPK/SIRT1/PGC-1α activation counteracts oxidative condition. PPARs are other common targets of SIRT1/AMPK: PPARα activation is related to inflammation, PPARγ, interacting with PGC-1α, improves adipose tissue plasticity and adipose browning tissue. Finally, PPARδ upregulation improves glucose metabolism in skeletal muscle.

Data reported in literature have shown that AMPK, through increasing NAD^(+)^ cellular levels, promotes the activity of Sirtuin 1 (SIRT1), which is a crucial NAD^(+)^-dependent histone deacetylase implicated in numerous cellular process from cell metabolism to cell growth [42,43,44]. Interestingly, AMPK and SIRT1 synergically act: Liver Kinase B1, a crucial upstream AMPK activator, is a main SIRT1 target [45]. Moreover, AMPK and SIRT1 have many common molecular targets involved in oxidative and inflammatory processes characterizing cardiometabolic pathologies, i.e., endothelial nitric oxide (NO) bioavailability, PCG-1α, and PPARs [38,39,40,41,42,46,47] (Figure 2). 

AMPK/SIRT1 action is related to endothelial nitric oxide (NO) bioavailability, which is significantly decreased in diabetic or cardiac patients [48,49], and it is correlated not only with endothelial function but also with oxidative and immune mechanisms [50]. Even if accumulating data have pointed out that diets rich in green leafy vegetables represent an important source of NO [51], the endothelial nitric oxide synthase (eNOS) enzyme is primary involved in NO production from the amino acid L-arginine [52]. It is well established that excessive oxidative stress impairs eNOS activity while NOX (Nicotinamide Adenine Dinucleotide Phosphate (NADPH) oxidase) enzymes, which are mainly responsible for ROS cellular generation, are upregulated [48,53]. In obesity or in a hyperglycemic state, ROS rapidly reacts with NO, forming secondary reactive nitrogen species, including peroxynitrite, thus decreasing NO bioavailability. Unbalanced NO production exacerbates stress conditions, creating a vicious cycle that mainly causes vasodilation damages and increases low-grade inflammation. A growing body of evidence has indicated that CR ameliorates endothelial dysfunction, activating AMPK-eNOS signaling [54,55]. Different studies have demonstrated that AMPK increases eNOS expression in an indirect manner by the phosphatidylinositol-3-kinase-protein/AKT pathway [56,57]. In addition, it has recently been demonstrated by García-Prieto et al. that Ca^2+^/calmodulin-dependent kinase II plays a crucial action in mediating CR-induced AMPK activation through H_2_O_2_ increase in aortas from obese rats [58]. In addition, AMPK improves NO bioavailability by downregulating NOX4 expression, as demonstrated by studies using different drugs able to active AMPK [59]. For instance, Hasan et al. have recently demonstrated that canagliflozin, a sodium–glucose cotransporter 2 (SGLT2) inhibitor used in T2DM treatment, ameliorates a cardiac oxidative microenvironment by AMPK/NOX4 signaling [60]. In the same manner, SIRT1 plays a fundamental role in eNO expression. In vivo studies have shown the interplay between SIRT1 and eNOS: CR actives SIRT1 that deacetylates and activates eNOS, while the acetylation of eNOS downregulates SIRT1 signaling [61]. Moreover, AMPK/SIRT1 activation enhancing eNOS signaling counteracts ischemia/reperfusion [62]. Finally, the closed relationship between SIRT1 and NOX is well recognized. Luo et al. have proven that dulaglutide, a common drug used in TDM2 treatment, activates SIRT1 and thus represses NOX4 expression in human umbilical vein endothelial cells [63]. 

PGC-1α, the primary factor involved in mitochondrial biogenesis [64], is activated by SIRT1 removing the acetyl groups, while AMPK-induced activation is mediated by a phosphorylation mechanism. AMPK/SIRT1/PGC-1α action on mitochondrial biogenesis increases the expression of anti-oxidation genes, mitigating oxidative microenvironment and cardiac damages [35,65]. The activation of the SIRT1/PGC-1α axis has been reported also by Waldman et al., who have observed an improvement of diabetic cardiomyopathy in db/db mice by CR diet treatment associated with a significant enhancement of oxidative stress and inflammation state [66]. Moreover, recently, Mehrabani et al. have speculated that CR could play an important role in preserving the normal homeostasis of the mitochondria population, enhancing mitophagy [42], as has been observed by Gutierrez-Casado et al. in a murine model [43]. This action is correlated with the Fork Head Box O1 (FOXO) transcription factor family. In particular, FOXO1 coordinates the expression of the primary enzymes involved in ROS scavengers and is deacetylated by SIRT1 in a dependent or independent manner of AMPK activation [67,68,69].

Peroxisome proliferator-activated receptors (PPARs) are other common targets of SIRT1/AMPK. PPARs interact with different co-regulators, including FOXO and PGC1-α, and influence several cellular functions, i.e., cellular metabolism, skeletal muscle and adipose tissues differentiation, inflammation, and oxidative stress. PPARα is involved in oxidative and inflammatory process. Kauppinen et al. have observed that PPARα, activated by SIRT1, inhibits NF-kB pathways and alleviates the inflammatory storm induced by hyperglycemia and lipotoxicity [70]. In addition, CR action on AMPK-PPARα is associated with a reduced monocyte mobilization and consequently with an improvement of chronic inflammatory state [71]. In obese mice affected by cardiomyopathy, CR promotes PPARα expression in the heart, reducing inflammation [72]. PPARγ is another member of the PPAR family that is primary involved in adipose tissue remodeling [73]. As is known, the modulation of adipose plasticity is a key mechanism to prevent cardiovascular complications in obese and diabetes patients, since white adipose tissue positively relates with high cardiometabolic risk, while brown adipose tissue and beige adipose tissue are negative correlated with cardiovascular risk [74]. PPARγ is activated by AMPK [75] or by SIRT1-mediated deacetylation [76] and interacts with PGC-1α, promoting the expression of mitochondrial uncoupling protein-1 expression (UCP1) and the recruitment of PRDM16, the main transcription factors involved in white adipose tissue browning [74,76,77,78]. In addition, PPARγ–PGC1α upregulates UCP2 expression, which affects mitochondrial dysfunction and ROS accumulation [79]. Different data corroborate CR action on PPARγ–PGC1α [80,81,82]. Finally, AMPK and SIRT1 also regulates PPARδ expression [83,84]. This member of the PPAR family is high expressed in skeletal muscle and is involved in glucose metabolism [85]: its activation improves glucose oxidation and exercise performed [83,86]. 

Moreover, accumulating recent data suggest that CR contributes to maintain the health of the intestinal epithelial barrier and then counteracts the gut stress/inflammation process. Indeed, CR action decreases LPS production and modifies microbiota composition [87,88]. A growing number of data have pointed out that CR-induced microbiota improvement is correlated not only with mitigated hepatic lipid accumulation [89,90] but also with increased fat adipose plasticity [73,91,92]. It is important to highlight that Correles et al. have demonstrated how, in a murine model, long-term CR has a strong impact on adipose plasticity, improving subcutaneous white adipose tissue expandability and the thermogenesis process of brown adipose tissue [92], as reported above, by PPARγ–PGC1α activation [73,82]. 

Therefore, even if CR dietary protocol, activating fundamental metabolic and antioxidant pathways, is able to guarantee weight loss, but in daily clinical pratice its effectiveness is limited by the adherence of patients [93]. Numerous subjects follow a diet program for a few months and usually recover weight loss. To minimize this problem, several investigations have been carried out aimed at identifying easy-to-administer CR mimetics [94,95,96,97]. Different drugs, including metformin and aspirin, have been defined CR mimetics [40,97], but food bioactive molecules arouse greater attention considering their easier use.

## 2. From Caloric Restriction to CR-Related Nutrients: Berberine

Berberine (BBR) is a component of many plants, i.e., Barberry, Berberis, Coptis chinensis, and Hydrastis, which is usually used in Chinese traditional medicine as an antibacterial drug [98]. 

The CR mimetic action of BBR has been established by numerous studies performed using in vitro and in vivo models of obesity or diabetes or cardiovascular pathological conditions [99,100]: BBR, activating mainly AMPK, reduces body weight and hepatic lipid accumulation, and it also improves insulin action [100,101,102,103,104]. Wu et al. have recently demonstrated that BBR not only increases glucose metabolism and the insulin signal pathway but also decreases inflammatory response in hepatocytes cultured in an insulin-resistant condition [105]. In obese and diabetic animal models, BBR treatment is able to enhance the AMPK–PGC-1α signaling pathway, mitigating the oxidative and fibrosis process caused by excessive adipose depots [106]. Moreover, it has been proven that in adipose tissue, SIRT1 is a main regulator of the insulin-sensitizing action of BBR [107]. BBR-induced SIRT1 activation is involved not only in the metabolic state but it also alleviates the inflammation state, decreasing the production inflammatory cytokines and macrophage infiltration [107]. Finally, as previous reported, AMPK activation is related to adipose tissue remodeling, and indeed, BBR promotes brown adipose tissue thermogenesis and white adipose tissue browning [108]. 

Further studies have highlighted BBR action on heart: Using an in vitro model of hyperglycemic cardiomyocytes, Hang et al. have observed that BBR, activating AMPK signaling, reduces ischemia/perfusion damages [109], and Chan et al. have confirmed these findings in a diabetic rat model [110]. As CR, BBR counteracts hyperglycemia-induced endothelial dysfunction through AMPK/eNOS signaling cascade activation [111]. 

Moreover, different authors have investigated BBR action on gut microbiota: BBR treatment modifies gut composition in animal models of obesity, alleviating the inflammation state induced by LPS overproduction and improving energy metabolism and insulin resistance condition [112,113,114]. In addition, in a mice model of atherosclerosis fed a high-fat diet, the modifications of microbiota composition induced by BBR are correlated with a significant decrease of inflammatory cytokine expression and an improvement of atherosclerosis state [115]. 

Taken together (Table 1), these results indicated that BRR acts in a similar manner to CR, ameliorating cardiometabolic condition as underlined by different systematic reviews and a meta-analysis of randomized controlled trials [116,117,118].

## 3. From Caloric Restriction to CR-Related Nutrients: Resveratrol

BBR is not the only food compound having CR mimetic propriety; the best-known food molecule characterized by CR mimetic action is certainly resveratrol (RSV). RSV is a natural polyphenolic compound processed by several plants and found in certain fruits, including peanuts, berries, and grapes [119]. At the beginning, interest for RSV has been linked to epidemiological studies aimed at clarifying cardioprotection effects of red wine [120]. Subsequently, in numerous investigations performed in vitro and in animal models, it has been established that RSV mainly (i) regulates high blood pressure and ameliorates vascular biology [121]; (ii) counteracts NAFLD progression [122]; (iii) improves insulin sensitivity [123]; (iv) promotes adipose tissue remodeling [124]; and (v) modifies gut microbiota composition [125], acting on the same mechanism activating by CR. 

RSV reduces high blood pressure, enhancing the AMPK–SIRT1 axis [126]. Moreover, RSV increases nitric oxide and simultaneously decreases ROS production [127,128] (Table 2).

In hepatocytes affected by steatosis or in NALFD mice models, RSV thwarts hepatic steatosis, dropping hepatic lipid accumulation and inflammation by AMPK/SIRT1 activation [129,130,131]. Teng et al. have observed that in steatotic hepatocytes (HepG2 cell model), RSV treatment decreases triglyceride accumulation by modulating the AMPK/SIRT1 signaling pathway. In addition, in mice affected by NAFLD, RSV not only counteracts liver steatosis but also recovers hepatic insulin sensitivity [132] (Table 2). 

RSV improves insulin sensitivity not only in liver but also in adipose tissue and in skeletal muscle. Chen et al. have observed how through acting on the SIRT1/AMPK axis, RSV improves the insulin pathway and glucose translocation in adipocytes [133]. In insulin-resistant skeletal muscle cells, AMPK activation by RSV increases insulin-mediated GLUT4 translocation [134]. Finally, Shu et al. have proved through in vitro and in vivo models of insulin resistance that RSV increases microRNA (miRNA) mmu-miR-363-3p levels and consequently improved AKT signaling, ameliorating metabolic condition [135] (Table 2).

Adipose tissue remodeling represents a further common action between RSV and CR. RSV-treated obese rats or mice are characterized by a significant reduced adipose tissue mass and an increased thermogenesis associated with high expression of SIRT1 and UCP1 [136]. In addition, mice fed a high-fat diet with 0.1% RSV have shown a significant increase of AMPK activation and an upregulation of brown adipocyte markers, including UCP1 [137]. Finally, Hui et al. have demonstrated that 10 weeks of RSV treatment promotes glucose metabolism and the adipose browning process not only by AMPK activation but also by gut microbiota composition modification, as demonstrated by antibiotic treatment. The depletion of gut microbiota, caused by antibiotics, partially abolishes RSV effects [138]. Data obtained by this work strengthen accumulating evidence that reveals how RSV modifying gut microbiota leads to weight loss, insulin sensitivity improvement, and white adipose tissue conversion in beige adipose tissue [139]. In addition, RSV action on gut mitigates oxidative stress and inflammation [140]. Furthermore, current studies suggest that RSV, via gut microbiota remodeling, attenuates the atherosclerosis process and increases hepatic bile acid neosynthesis, downregulating the enterohepatic farnesoid X receptor-fibroblast growth factor 15 axis [141]. In addition, RSV counteracts atherosclerosis onset action on endothelial dysfunction, as is well known. RSV’s endothelial positive effects are mediated by AMPK/SIRT1/eNOS signaling cascade in a similar manner to that of CR [142].

Unfortunately, the efficacy of RSV supplementation as a CR mimetic is not yet confirmed by clinical studies: a small number of clinical trials have been performed, and the results are discordant [143,144]. It is important to remember that RSV bioavailability is very low, and several authors have observed how different RSV doses are correlated with different actions [145,146]. Identifying RSV effective dose and pharmacokinetics probably represents the crucial question to solve [147]. 

## 4. From Caloric Restriction to CR-Related Nutrients: Quercetin

Quercetin (QE), an important member of flavonoids principally found in elderberries, onions, cranberries, apples, and in other fruits and vegetables is one of well-known dietary antioxidant compounds [148]. As BRR and RSV in vitro and in vivo studies show, QE ameliorates hypertension, heart failure condition [149], obesity, diabetes, inflammation state [150], and gut dysbiosis [151]. 

Accumulating lines of evidence indicate that QE plays an important anti-hypertensive action mainly through increasing nitric oxide (NO) production, decreasing oxidative stress, and activating the AMPK signaling pathway, as observed by Calabrò et al. in hypertensive rats [152]. In addition, Kim et al., using an in vitro model, have observed that QE activating AMPK signaling attenuates vascular smooth muscle cells contraction [153]. Moreover, QE treatment improves cardiac hypertrophy, which is a pathological cardiac modification that usually leads the onset of heart failure [154,155,156]. In an in vitro model of rat cardiomyocytes, QE action is mediated by PPARγ [154], while Guo et al. have proven that QE counteracts hypoxic damages and prevents cardiomyocytes apoptosis by AMPK–SIRT1 axis activation [156]. Then, QE also activates the same molecular mediators as CR. 

As previous reported, a hyperglycemic-induced oxidative microenvironment is a crucial cause of therapeutically ineffective cardioprotection interventions. Roslan et al. have observed that QE treatment alleviates metabolic alteration, increasing insulin levels and contemporarily decreasing cardiac inflammatory and oxidative stress markers [157]. QE action on inflammatory mediators has been also observed in the liver of obese/diabetes mice [158,159]. Zhu et al. have studied QE action in an in vitro and in vivo model, demonstrating that QE reduces hepatic triglyceride and promotes lipophagy [160]. Moreover, different works have reported that isoquercetin, a QE derivative, activates AMPK alleviating hepatic lipid accumulation [161,162]. In addition, in skeletal muscle, QE enhances AMPK/SIRT1 signaling cascade and promotes glucose uptake [163,164,165]. Moreover, other data have revealed that QE stimuli regulate IRSs phosphorylation, alleviating insulin resistance and promoting pro-oxidant/antioxidant enzymes balance [166,167]. 

As CR, QE influences adipose tissue. As studied by Forney et al., QE inhibits lipid accumulation, decreasing adipose tissue mass [168]. In addition, the adipose-induced inflammation process is decreased by QE. Researchers have not completely clarified the molecular mechanisms used by QE to counteract adipose inflammation; probably, QE activates MAPK kinases cascade and downregulates NOX enzymes [169,170]. Recently, different groups have observed that QE improves the inflammasome response, ameliorating gut dysbiosis [171]. In addition, in this case, QE action on the gut is associated with a reduced atherosclerotic process [172,173]. In detail, Zhang et al. have observed that QE treatment in obese diabetic rats improves the lipid metabolism and reduces the number of atherosclerotic lesions, enhancing the activity of antioxidant enzymes in the carotid artery. They demonstrated, using a specific AMPK inhibitor (compound-C), that QE mitigates atherosclerotic damages, activating the AMPK/SIRT1/NF-κB signaling pathway [174]. 

Finally, it is important to remember that not only QE but also its metabolites, such as rutin, play a positive role in diabetes and cardiovascular diseases [149,175]. Taken together, these results indicate that QE promotes cardiometabolic health, activating several common molecular targets of CR (Table 3). 

However, the data collected about the effects of QE are partially conflicting, and as with RVS, pharmacokinetics is the main problem to solve [149,150,151,176]. 

## 5. From Caloric Restriction to CR-Related Nutrients: L-Carnitine 

In recent years, several works have shed new light on L-carnitine (LC), which is the biologically active form of dietary nutrient carnitine found principally in red meat and eggs [177]. 

Over the past decade, several research groups have demonstrated a positive correlation between high atherosclerosis risk and trimethylamine-N-oxide (TMAO), which is produced by gut bacterial metabolism of betaine and above all of choline and LC [178]. Several data reported in the literature have shown that TMAO activates the NF-κB pathway, enhancing the production of inflammatory cytokines and chemokines besides increasing ROS levels [179,180,181]. Moreover, TMAO is associated with an unbalanced regulation of cholesterol production and, in detail, with an impaired reverse cholesterol transport to and from the liver and small intestine [182,183,184]. Numerous data have indicated that LC supplementation accelerates TMAO-induced atherosclerosis in mice models [185,186]. Recently, Bordoni et al. observed that in aged women, LC intake and consequently TMAO production cause epigenetic alterations of mitochondrial DNA in platelets, contributing to atherosclerosis development [187]. Recently, epidemiological studies have established that only concomitant high levels of plasma LC and TMAO are associated with an increased risk of CVD development [188,189]. 

This simple overview would suggest limiting carnitine intake, but several other data indicate that LC plays, through AMPK signaling, an important cardioprotective role, counteracting mitochondrial dysfunction and aberrant ROS synthesis.

LC supplementation ameliorates left ventricular dysfunction, decreasing ROS production and improving glucose and fat acid metabolism [190]. Moreover, several authors have proven that LC stimuli mitigate ischemia–reperfusion-induced damages, activating the principal cellular mechanisms involved in anti-oxidative and anti-apoptotic mechanisms, mainly reperfusion injury salvage kinase (RISK) and survivor-activating factor enhancement (SAFE) signaling pathways [191,192,193]. Additionally, our group recently demonstrated the cardioprotective LC action in an in vitro model of hyperglycemic cardiomyocytes [194], and above all, we have proven that LC effects are mediated by AMPK. Then, LC supplementation could be used in patients affected by chronic heart disease, such as left ventricular dysfunction. Moreover, in acute conditions, such as in myocardial infarction, LC could mitigate abnormal oxidative stress condition and enhance cardiomyocytes survival. Animal studies and preliminary clinical studies seem to confirm this hypothesis [195,196,197,198]. It is important to note that these first trials suggest that LC improves antioxidative cellular defense, in particular the NRF2 pathway [198]. 

Different results have shown that LC implementation has not only antioxidant action but also hypoglycemic effects in diabetic rats [199]. In addition, several works have reported that LC ameliorates NAFLD condition decreases lipid accumulation and prevents NAFLD-induced heart dysfunction. The possible mechanism of this LC action is related to its ability to activate redox-signaling pathways, promoting mitochondrial activities and AMPK signaling [200,201]. In confirmation of the AMPK role in LC action, our group recently demonstrated that LC supplementation mitigates liver steatosis induced by fructose, activating AMPK and consequently increasing anti-oxidative cellular response [202]. Similarly, Sayed-Ahmed et al. have observed that LC supplementation mitigates chemotherapy-induced cardiotoxicity, increasing AMPK signaling [203].

As reported above, LC intake is associated with TMAO production, and this is a crucial limit on LC use in the treatment of cardiometabolic pathologies. Zhao et al. have demonstrated, in a mouse model of atherosclerosis (ApoE^−/−^ mice), that subcutaneous LC administration, bypassing intestinal-liver TMAO formation, does not aggravate the atherosclerosis process [204]. Then, it is hypothesized that the synergic use of LC and drugs capable of inhibiting TMAO synthesis could represent the ideal solution. TMAO is primary produced following an oxidative reaction of microbial metabolite trimethylamine (TMA) catalyzed by hepatic flavin monooxygenase 3 (FMO3) [183]. FMO3 knockout mice and subjects with a genetic defect in FMO3, despite having higher levels of TMA, are not characterized by a higher risk of atherosclerosis [205,206]. Then, modulating FMO3 expression, it is possible to modify TMAO production. Different studies have demonstrated that enterohepatic farnesoid X Receptor (FXR), a nuclear receptor, enhances FMO3 expression [206], and natural compounds (RSV) [141,207] or chemical antagonists of FXR (guggulsterone) [208] positively modulate the TMAO/TMA ratio. Therefore, further studies are needed to explore this therapeutical opportunity to resolve the TMAO “problem” and to use LC supplementation as mimic caloric restriction (Table 4). 

## 6. From Caloric Restriction to CR-Related Nutrients: Bioavailability and Pharmacokinetics 

As mentioned above, while obtained data from in vitro and in vivo studies are very encouraging for a possible use of BBR, RSV, and QE and LC in management of diabetes and cardiovascular diseases complications, clinical data are partly controversial. Bioavailability, dose, and pharmacokinetics are fundamental aspects that have yet to be completely clarified, and this limits the use of these nutraceutical compounds [147,176]. 

To solve these problems, some authors have proposed the use of synthesized products having similar proprieties of nutraceuticals. SRT2104, a SIRT1 activator as RSV, is one of the best known of these compounds [209,210,211]. Unfortunately, even using SRT2104, the results of small clinical studies are partly conflicting. Baksi et al. performed, for 28 days, a phase II, randomized, double-blinded study of SRT2104 in diabetics subjects. They used different doses of SRT2104 but did not observe a positive significant impact on metabolic parameters (no effect on glycemia and HbA1c, modest action on lipid), but the results of their study shown above were characterized by a highly variable response [209]. Meanwhile, Noh et al., studying in the same manner of 28 days of SRT2104 treatment in diabetic patients, observed significant weight loss and an improvement of glycemic control but neutral impacts on endothelial and fibrinolytic function [210]. This last finding is in contradiction with the results previously obtained by the same research group in diabetic patients and healthy smokers [211]. This brief overview points out the main problems associated with the development of synthetic compounds capable of mimicking RSV action: the small size of analyzed groups and subjects’ variability constitute an important limitation to clarifying the action and the pharmacokinetics of SRT2104 and other similar innovative drugs [212]. 

Similarly, to improve BBR bioavailability, many derivatives of BBR were synthesized. For example, dihydroberberine (dhBBR) and 8,8-dimethyldihydroberberine (Di-MeBBR) reduce the atherosclerotic process in ApoE^−/−^ mice [213]. Wang et al. have designed and synthesized four series of BBR derivatives that have potent hypoglycemic activity due to the AMPK activation pathway [214]. However, even then, further investigations should be performed to clarify molecular mechanisms.

To strengthen the effects of CR mimetics compounds, different research groups are investigating the synergic use of drug and food CR mimetics or two CR mimetics, obtained encouraging results [215,216,217,218]. However, even these studies are often contradictory. 

So perhaps a possible interesting solution could be represented by the development of the nutritional strategies based on new CR regimens, i.e., intermittent fasting regimes and time-restricted feeding, and CR mimetics. An intermittent fasting regime is characterized by alternate periods of fasting with recurring periods, usually 1 or 2 fasting days per week or on alternative days [219]. A time-restricted program requires that food consumption be limited to certain hours of the day [220]. These regimes seem to have a similar effect to CR but do not require ongoing commitment from patients; therefore, these diet strategies could be used to overcome CR problems linked to patients’ adhesion, and food CR mimetics could be used to strengthen their effectiveness.

## 7. Conclusions 

CR, an emerging restrictive nutritional approach, enhances healthspan and lifespan through ameliorating metabolic and cardiovascular functions and decreasing oxidative and low-grade inflammation states. The AMPK/SIRT1 signaling cascade is the key molecular pathway influenced by CR. AMPK/SIRT1 activation is associated with (i) increased mitochondrial and endothelial function, (ii) improved gut dysbiosis, (iii) ameliorated muscle and hepatic insulin signaling, (iv) and increased adipose tissues remodeling. However, for most subjects, performing this nutritional regime is impossible. Caloric restriction is not only a diet, but an important change of lifestyle, and it is a challenge for many patients affected by T2DM and/or CVD who usually are unable to observe these restriction conditions for a long period. 

For this reason, different research groups have identified and studied different nutritional compounds capable of mimicking caloric restriction effects. Berberine, resveratrol, and quercetin are the best-known CR mimetics characterized by their action on AMPK/SIRT1 signaling. Moreover, recent data have shown that LC could be used in the management of diabetes and cardiovascular diseases as well. 

**Figure 3:**
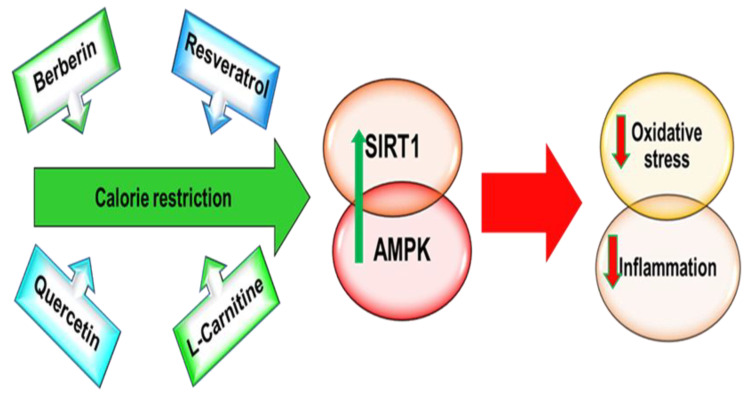
CR-related nutrients. Calorie restriction through AMPK/SIRT activation reduces the risk of developing T2DM and CVD. Berberin, resveratrol, quercetin and L-carnitine are also capable of activating AMPK/SIRT1 and therefore could be used as CR mimetics to preserve a healthy cardiometabolic state.

Then, the consumption of these nutraceuticals or innovative functional food enriched with these nutritional components could represent an important nutritional strategy in T2DM and CVD management (Figure 3).

In addition, it is important to note that AMPK is the key molecular regulator of exercise action [221]. Moreover, some researcher groups, including us, have demonstrated how CR mimetics, for instance RSV and LC, enhance skeletal muscle differentiation [146,222]. Therefore, it appears essential to study the combined effect of nutritional interventions based on CR mimetics and exercise. In the future, identifying nutritional agents able to improve cardiometabolic state should be evaluated in associated with lifestyle therapeutic interventions (diet–exercise). Above all, as mentioned before, pharmacokinetic aspects related to use of nutraceutical molecules still need to be fully clarified, and before usage on a large scale, they should be analyzed in different clinical trials focused on lifestyle therapy. 

In conclusion, T2DM and CVD are the principal global health threats of the future decades, and new dietetic regimes based on caloric restriction help to prevent the onset of these pathologies. However, many subjects fail to follow these dietary protocols, and identifying nutraceuticals capable of activating the same metabolic pathways of CR should be fundamental. 

In this regard, berberine, resveratrol, quercetin, and L-carnitine have demonstrated CR properties. Nevertheless, the heterogeneity of data obtained using different doses of these dietary bioactive compounds represents the most crucial limit to propose specific recommendations. Moreover, current data show the importance of synergic action of diet and exercise and highlight the need to study the CR mimetics effects in relation to lifestyle therapeutic interventions (diet–exercise) to define nutritional recommendations for patients.

## Figures and Tables

**Table 1:** Berberine as a CR-related nutrient (↑ increase/improvement, ↓ decrease/worsening).

Type of Studies	Tissue Molecular Mechanisms	Effects	References
In vitro and in vivo (obese mice)	Skeletal muscle: ↑ AMPK/PGC-1α pathway activation	↓ lipid deposition in skeletal muscle ↑ glucose metabolism ↑ mitochondrial biogenesis and function	Yao et al. [103]
In vitro study	Hepatocytes (HepG2): ↑ AMPKα1 activation in	↑ glucose and lipid metabolism	Ren et al. [104]
In vitro and in vivo (diabetic rats)	Liver: ↑ PKA activation	↓ inflammatory response	Wu et al. [105]
In vivo (obese rats)	Adipose tissue: ↑ AMPK activation	↓ body weight ↑ glucose metabolism ↓ fibrosis response in adipose tissue	Wang et al. [106]
In vitro and in vivo (obese rats)	Adipose tissue: ↑ AMPK/SIRT1/PGC-1α activation	↑ insulin sensitizing ↓ inflammation state ↓ macrophage infiltration	Shan et al. [107]
In vivo (obese mice) and clinical study (overweight NAFLD patients)	Brown adipose tissue: ↑ AMPK/PRDM16 signaling cascade	↑ activation of brown adipose tissue	Wu et al. [108]
In vitro	Cardiomyocytes grown in high glucose: ↑ AMPK/ activation	↑ mitochondrial biogenesis	Hang et al. [109]
In vivo (diabetic rats with cardiac ischemia)	Non-ischemic areas of the diabetic heart: ↑ AMPK activity	↓ damages induced by ischemia–reperfusion injury	Chang et al. [110]
In vitro	Cultured endothelial cells and blood vessels isolated from rat aorta: ↑ AMPK/eNOS signaling	↑ improved endothelial dysfunction ↑ vasodilatation	Wang et al. [111]
In vivo (obese rats)	Liver: ↓ Toll-like receptor 4 (TLR4)/tumor necrosis factor (TNF)-α pathway	↑ improved insulin resistance ↓ hepatic steatosis and LPS release	Liu et al. [112]
In vivo (Sprague–Dawley rats and hamsters, obese mice)	↑ Butyrate production by gut microbiota	↓ blood lipid and glucose levels	Wang et al. [113]
In vitro and in vivo (mice)	Gut microbiota: ↓ Clostridium species	activation of intestinal FXR	Tian et al. [114]
In vivo (obese male apoE^−/−^ mice)	Modification of gut composition	↓ atherosclerosis development, inflammatory cytokine expression, hepatic FMO3 expression and TMAO	Shi et al. [115]

**Table 2:** Resveratrol as a CR-related nutrient (N.D. = no date, ↑ increase/improvement, ↓ decrease/worsening).

Type of Studies	Tissue Molecular Mechanisms	Effects	References
In vivo (obese rats)	aortas: ↓ NOS signaling pathway	↓ endothelial dysfunction and vascular insulin resistance	Akar et al. [126]
In vivo (hypertensive rats)	rostral ventrolateral medulla (RVLM): ↑ AMPK activation	↓ blood pressure and ROS generation ↑ ERK1/2–RSK–nNOS pathway	Cheng et al. [127]
In vivo (hypertensive rats)	endothelium: ↑ superoxide dismutase activity	↓ oxidative stress induced by altered nitrite/nitrate levels ↓ development of hypertension	Bhatt et al. [128]
In vivo (obese rats)	liver: ↑ activation of SIRT1 signaling ↑ autophagy	↓ endoplasmic reticulum stress ↓ hepatic lipid accumulation	Ding et al. [129]
In vitro and in vivo (obese rats)	Liver and hepatocytes treated with high concentration of glucose and insulin: ↑ AMPK activation	↓ triacylglycerol (TG) accumulation ↑ improved insulin resistance	Shang et al. [130]
In vitro and in vivo (obese rats)	Liver: ↑ PKA/AMPK/PPARα signaling pathway activation	↓ redox homeostasis and lipid accumulation	Huang et al. [131]
In vitro and in vivo (NAFLD mice model)	Liver: ↑ AMPK/SIRT1/FAS/ SREBP1c signaling pathway activation	↓ triglyceride accumulation ↑ improved insulin resistance	Teng et al. [132]
In vitro	3T3 L1 adipocytes: ↑ SIRT1–AMPK signalling activation ↑ FOXO nuclear translocation	↑ glucose metabolism ↑ improved insulin resistance	Chen et al. [133]
In vitro	Skeletal muscle cells: ↑ AMPK activation	↑ GLUT4 translocation ↑ improved insulin resistance	Vlavcheski et al. [134]
In vitro and in vivo (obese mice)	Liver and hepatocytes: ↑ PI3K–Akt signalling activation	↑ improved insulin resistance	Shu et al. [135]
In vivo (obese mice) and human study (obese volunteers aged 30–55 years)	Adipose tissue: ↑ SIRT signalling activation	↑ improved glycemic and lipid profiles ↑ expression of genes (UCP1, PRDM16, PGC1α) involved in adipose tissue thermogenesis	Andrade et al. [136]
In vivo (obese female mice)	Adipose tissue: ↑AMPK activation	↑ brown-like adipocyte formation in inguinal white adipose tissue	Wang et al. [137]
In vivo (obese mice)	Adipose tissue and gut: ↑ gut microbiota–bile acid–TGR5/UCP1 pathway	↑ brown adipose tissue activation and white adipose tissue browning	Hui et al. [138]
In vivo (obese mice)	Adipose tissue and gut: ↑ SIRT1 signalling activation	↓ fat accumulation ↓ gut microbiota dysbiosis ↑ white adipose tissue browning	Liao et al. [139]
In vivo (obese mice)	Adipose tissue: ↑ antioxidative mitochondrial pathway	↓ body weight gain ↓ oxidative and inflammatory condition ↓ gut microbiota alterations	Campbell et al. [140]
In vivo (atherosclerotic mice model)	↓ enterohepatic farnesoid X receptor-fibroblast growth factor 15 axis	↑ gut microbiota remodeling ↑ hepatic bile acid neosynthesis ↓ TMAO production	Chen et al. [141]

**Table 3:** Quercetin as a CR-related nutrient (↑ increase/improvement, ↓ decrease/worsening).

Type of Studies	Tissue Molecular Mechanisms	Effects	References
In vivo (rats)	Heart: ↓ NADPH oxidase (NOX)-dependent superoxide anion production	↓ blood pressure ↑ activities of oxidant detoxifying enzymes	Calabrò et al. [152]
In vitro	vascular smooth muscle cells: ↑ AMPK activation	↓ myosin light chain kinase (MLCK) expression ↓ phosphorylated myosin light chain	Kim et al. [153]
In vitro and in vivo (hypertensive rats)	Heart and hypertrophic cardiomyocytes: ↑ PPAR-γ expression ↓ AP-1 signaling pathway	↓ blood pressure ↓ reduced the ratio of left ventricular to body weight	Yan et al. [154]
In vivo (hypercholesterolemic mice)	Blood sample	↓ total cholesterol and very low-density lipoprotein ↓ maladaptive myocardial remodeling	Ulasova et al. [155]
In vitro	Cardiomyocytes ↑ SIRT1–AMPK signaling pathway activation after hypoxia damages	↓ apoptosis	Guo et al. [156]
In vivo (diabetic rats)	Heart: ↑ activity level of cardiac anti-oxidative enzymes	↓ cardiac injury ↑ hemodynamic parameters ↑ metabolic profile	Roslan et al. [157]
In vivo (obese diabetic mice)	Liver: ↓ p65/NF-κB and ERK1-2/MAPK signaling pathways	↓ body weight gain, oxidative state, and liver injury ↑ metabolic profile	Zhang et al. [158]
In vivo (obese diabetic mice)	Liver: ↑ activity level of hepatic anti-oxidative enzymes	↑ metabolic profile and adiponectin serum level ↓ oxidative state and dyslipidaemia	Jeong et al. [159]
In vitro and in vivo (obese rats)	Liver and hepatocytes: ↑ IRE1a/XBP1s pathway signaling activation ↓ lipophagy	↓ hepatic steatosis	Zhu et al. [160]
In vivo (obese rats)	Liver: ↑AMPK activation ↓ TGF-β signalling	↓ lipid accumulation ↓ inflammation state ↓ oxidative stress	Qin et al. [161]
In vitro	Rat hepatoma cells (H4IIE): ↑ AMPK activation and AdipoR1 expression ↓ SREBP-1 and FAS expression	↓ lipid accumulation	Zhou et al. [162]
In vitro	Skeletal muscle cells, murine and human hepatocytes: ↑ AMPK activation ↑ GLUT4 translocation	↑ glucose metabolism	Eid et al. [163]
In vitro	Skeletal muscle cells: ↑ AMPK activation	↓ insulin-mediated glucose disposal in normal condition ↑ insulin resistance correlated to inflammatory condition	Liu et al. [164]
In vivo (obese diabetic rats)	Liver: ↑ SIRT1 expression ↑ AKT activation	↑ glucose and lipid metabolism ↓ hepatic histomorphological injury	Peng et al. [165]
In vitro	Endothelial cells: ↑ IRS1/PI3K signaling pathway activation ↑ Akt/eNOS signaling pathway activation	↓ inflammation state ↓ oxidative stress	Guo et al. [166]
In vitro	Hepatocytes: ↓ SREBP-1c and fatty acid synthase FAS	↓ hepatic lipid accumulation	Li et al. [167]
In vivo (obese mice)	Adipose tissue: ↓ inflammatory mediators	↓ adipocyte size and number in subcutaneous and visceral white adipose tissue	Forney et al. [168]
In vitro and in vivo (zebrafish and mouse)	Adipocytes and macrophages: ↓ adipogenic factors (C/EBPs and PPARγ) ↓ MAPK signaling pathway ↓ inflammatory cytokines	↓ weight gain ↓ lipid accumulation ↓ inflammatory state	Seo et al. [169]
In vivo (obese mice)	Adipose tissue: ↓ NFκB activity ↑ mitochondrial function	↓ inflammatory state in adipose tissue	Kobori et al. [170]
In vivo (obese mice)	Gut-liver: ↓ (TLR-4)-NF-κB signaling pathway	↓ intrahepatic lipid accumulation ↓ insulin resistance ↓ gut dysbiosis	Porras et al. [171]
In vivo (obese mice)	aortic sinus and gut microbiota	↓ atherosclerotic lesions and gut dysbiosis	Nie et al. [172]
In vivo (obese mice)	aortic sinus	↓ atherosclerotic lesions ↓ lipid accumulation ↑ microbiome diversity	Wu et al. [173]
In vivo (obese diabetic rats)	carotid artery: ↑ AMPK/SIRT1 activation ↓ NF-kB signaling pathway	↑ lipid profile ↓ atherosclerotic lesions ↓ oxidative stress	Zhang et al. [174]

**Table 4:** L-carnitine as a CR-related nutrient (↑ increase/improvement, ↓ decrease/ worsening).

Type of Studies	Tissue Molecular Mechanisms	Effects	References
In vivo (rats fed with choline deficient diet)	Heart	↑ cardiac function ↓ cardiac inflammation	Strilakou et al. [190]
In vivo (hypertensive rats)	Heart	↑ cardiac function ↓ blood pressure ↓ cardiac inflammation and fibrotic process	O’Brien et al. [191]
Human study (patients undergoing valve replacement)	Heart: ↑ Bcl-2 anti-apoptotic factor ↓ Bax pro-apoptotic factor	↓ cardiac cells apoptosis	Li et al. [192]
In vivo (mice with I/R injury)	Heart: ↑ PI3K/Akt activation ↑ Bcl-2 anti-apoptotic factor ↓ Bax pro-apoptotic factor	↑ myocardial contractile function ↓ myocardial apoptosis	Xue et al. [193]
In vitro	Cardiac cells (H9c2) grown in hyperglycemic condition: ↑ AMPK and STAT3 activation ↑ anti-oxidative factors	↓ oxidative stress	Vacante et al. [194]
In vivo (Sprague–Dawley rats with heatstroke-induced cardiac injury)	Heart: ↑ anti-oxidative factors	↓ inflammatory response ↓ oxidative stress ↓ cardiomyocytes apoptosis	Wang et al. [195]
In vitro and in vivo (rats with I/R injury)	Heart and cardiomyocytes: ↓ nuclear transcription-related factor 2/heme oxygenase-1 (Nrf2/HO-1)	↓ oxidative stress ↓ cardiomyocytes apoptosis	Zhao et al. [196]
Human study (meta-analysis)	↓ serum inflammatory mediators ↑ superoxide dismutase level	↓ inflammatory cytokines ↑ antioxidant mitochondrial enzymes	Fathizadeh et al. [197]
Human study	Heart: ↓ NF-κB signaling pathway ↑ Nrf2 levels	↓ inflammatory cytokines ↑ antioxidant mitochondrial enzymes	Li et al. [198]
In vivo (diabetic rats)	Skeletal muscle: ↑ anti-oxidative factors	↑ insulin sensitivity index ↑ metabolic profile ↑ contractile properties	Samir et al. [199]
In vivo (NAFLD model mice)	Heart and liver: ↓ hepatic NF-kB signaling ↑ hepatic PPARƔ ↓ myocardial ERK/STAT3 pathway	↓ hepatic steatosis ↓ hepatic fibrosis ↓ hepatic and myocardial oxidative stress	Mollica et al. [200]
Human study (meta-analysis in patients with NAFLD)	Liver	↑ hepatic function ↓ insulin resistance condition	Abolfathi et al. [201]
In vitro	Hepatic cells treated with fructose: ↑ AMPK activation	↓ lipid accumulation ↓ oxidative stress ↑ mitochondrial function	Montesano et al. [202]
In vivo (rats treated with sunitinib)	Heart: ↑ AMPK activation	↓ induced-sunitinib cardiotoxicity ↑ mitochondrial transport of LCFA	Sayed-Ahmed et al. [203]

## Abbreviations

T2DM	Type 2 diabetes mellitus
CVD	Cardiovascular diseases
LPS	Lipopolysaccharide
NAFLD	Non-alcoholic fatty liver disease
ROS	Reactive oxygen species
CR	Caloric restriction
AMPK	AMP-activated protein kinase
SIRT1	Sirtuin 1
NO	Nitric oxide
eNOS	Endothelial nitric oxide synthase
NOX	Nicotinamide Adenine Dinucleotide Phosphate-NADPH-Oxidase
FOXO	Fork Head Box O1
PPARs	Peroxisome proliferator-activated receptors
UCP1	Uncoupling protein-1 expression
BBR	Berberine
RSV	Resveratrol
QE	Quercetin
LC	L-Carnitine
TMAO	Trimethylamine–N-oxide
TMA	Trimethylamine
FMO	Flavin monooxygenase enzymes
